# POD1UM-303/InterAACT 2: A phase III, global, randomized, double-blind study of retifanlimab or placebo plus carboplatin–paclitaxel in patients with locally advanced or metastatic squamous cell anal carcinoma

**DOI:** 10.3389/fonc.2022.935383

**Published:** 2022-08-24

**Authors:** Sheela Rao, Mark Jones, Jill Bowman, Chuan Tian, Jean-Philippe Spano

**Affiliations:** ^1^ The Royal Marsden Hospital NHS Foundation Trust, London, United Kingdom; ^2^ Incyte Corporation, Wilmington, DE, United States; ^3^ APHP-Sorbonne University-IUC, Paris, France

**Keywords:** anal cancer, carboplatin, paclitaxel, retifanlimab, squamous carcinoma

## Abstract

**Background:**

Squamous carcinoma of the anal canal (SCAC) is a human papillomavirus (HPV)-driven cancer with poor prognosis in locally advanced or recurrent settings. Carboplatin–paclitaxel is the preferred first-line regimen for unresectable locally advanced or metastatic SCAC, with the reported median progression-free survival (PFS) and overall survival (OS) of 8.1 and 20.0 months, respectively. Immune checkpoint blockade (ICB) demonstrates improved survival in HPV-driven cervical and head and neck cancers. Retifanlimab (INCMGA00012) is an investigational humanized, hinge-stabilized, immunoglobulin G4κ monoclonal antibody targeting programmed cell death-1 (PD-1), with characteristics common to the ICB class. In POD1UM-202, retifanlimab showed substantial clinical activity and an expected safety profile in patients with advanced SCAC who progressed on platinum-based chemotherapy. Based on these encouraging results, POD1UM-303/InterAACT 2 (NCT04472429), a phase III, double-blind, randomized, multiregional study, investigates the addition of retifanlimab to the standard of care (SOC) carboplatin–paclitaxel in patients with inoperable locally recurrent or metastatic SCAC not previously treated with systemic chemotherapy.

**Methods and analysis:**

Patients ≥18 years with inoperable locally recurrent or metastatic SCAC, measurable disease per RECIST v1.1, and no prior systemic chemotherapy or PD-(L)1-directed therapy will be enrolled and stratified by PD-L1 expression, region, and extent of disease. Patients with well-controlled human immunodeficiency virus infection are eligible. Planned enrollment is approximately 300 patients worldwide, with a 1:1 randomization to retifanlimab or placebo. Patients will receive up to six induction cycles (24 weeks) of carboplatin (area-under-the-curve 5 on day 1) and paclitaxel (80 mg/m^2^ on days 1, 8, and 15) every 28 days per SOC. Concurrently, retifanlimab 500 mg or placebo will be administered intravenously in a blinded fashion on day 1 of each 28-day cycle for up to 13 cycles (1 year) in the absence of unacceptable toxicity, disease progression, withdrawal of consent, loss to follow-up, or premature discontinuation. Crossover to open-label retifanlimab will be allowed for patients assigned to placebo upon verification of progression by blinded independent central radiographic review (BICR). The primary study endpoint is PFS per RECIST v1.1 by BICR. Secondary endpoints are OS, objective response rate, duration of response, disease control rate, safety, and retifanlimab pharmacokinetics. The study is currently recruiting.

**Clinical Trial Registration:**

https://clinicaltrials.gov/ct2/show/NCT04472429; https://clinicaltrialsregister.eu/ctr-search/search?query=2020-000826-24

## 1 Introduction

Anal cancer is a rare malignancy accounting for 2.6% of approximately 1.9 million global cases of colorectal cancer in 2020, and of the almost 51,000 cases reported, 57% occurred in women (1). Squamous carcinoma of the anal canal (SCAC) is the predominant tumor type (80%) in anal cancer (2, 3). Epidemiological studies have reported an increasing incidence of SCAC in Europe and North America during the period 1989–2007 (4) and in the United States for 2001–2015 (5). The risk factors for SCAC include sexual practices (e.g., anal receptive intercourse, lifetime number of partners), history of sexually transmitted diseases, vulvar or cervical carcinoma/dysplasia, chronic immunosuppression [e.g., related to human immunodeficiency virus (HIV) or subsequent to organ transplantation], and smoking (6–8).

Chemoradiation is the standard of care (SOC) for localized SCAC, with a reported 5-year disease-free survival of 57.8% with cisplatin–fluorouracil plus radiotherapy (RT) and 67.8% with mitomycin–fluorouracil plus RT (9). Carboplatin–paclitaxel is currently the preferred first-line regimen for unresectable locally advanced or metastatic SCAC, with the reported median progression-free survival (PFS) and overall survival (OS) of 8.1 and 20.0 months, respectively (10).

The dominant etiology of SCAC is human papillomavirus (HPV) infection (6, 7, 11), with approximately 90% of tumors found to be HPV-positive (2, 12, 13). Immune checkpoint blockade (ICB) with programmed cell death (ligand)-1 [PD-(L)1] inhibitors is a promising approach to HPV-driven malignancy based on clinical experience in head and neck squamous cell carcinoma (HNSCC) (14) and cervical cancer (15).

Retifanlimab (INCMGA00012) is an investigational humanized, hinge-stabilized, immunoglobulin G4κ monoclonal antibody targeting PD-1, with characteristics common to the ICB class (16–19). Retifanlimab monotherapy demonstrated encouraging clinical efficacy in the treatment of 94 patients with advanced SCAC who progressed on or during a platinum-based regimen; the single-arm phase II POD1UM-202 study achieved an overall median PFS and OS of 2.3 and 10.1 months, respectively (20). Thirteen (13.8%) patients achieved an objective response (one complete response, 12 partial responses) and the median duration of responses was 9.5 months (range, 5.6–not estimable) (20). Thirty-three (35.1%) patients had stable disease, resulting in an overall disease control rate of 48.9%. Importantly, responses in the POD1UM-202 study were observed regardless of PD-L1 expression, liver metastases, or HIV status; retifanlimab was well-tolerated with a low incidence (2.1%) of treatment discontinuation because of immune-related adverse events (20). Retifanlimab response in locally advanced and/or metastatic SCAC results is supported by the results with other single-agent PD-(L)1 inhibitors (21–24), with the reported median PFS and OS of 4.1 and 11.5 months for nivolumab (21), 2.0 and 13.9 months for avelumab (24), and up to 3.0 and 11.9 months for pembrolizumab (22, 23), respectively.

Based on the encouraging results seen with retifanlimab in the platinum-refractory SCAC patient population in POD1UM-202, the phase III POD1UM-303/InterAACT 2 study was designed to examine the addition of retifanlimab to SOC in chemotherapy-naive patients with advanced SCAC.

## 2 Methods and analysis

### 2.1 Study design

**Figure 1:**
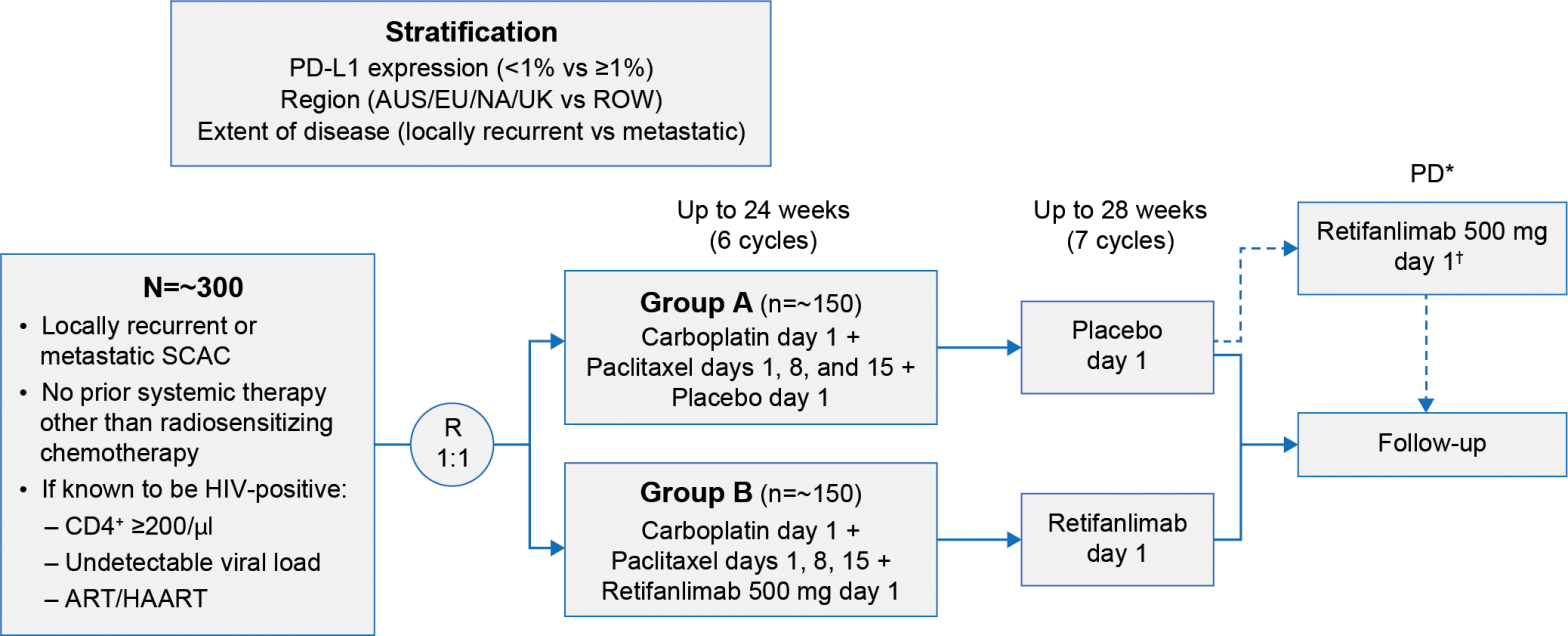
POD1UM-303/InterAACT 2 study design. *Verified by BICR. ^†^Optional crossover period for qualified patients. ART/HAART, antiretroviral therapy/highly active antiretroviral therapy; AUS, Australia; BICR, blinded independent central radiographic review; EU, European Union; HIV, human immunodeficiency virus; NA, North America; PD, progressive disease; PD-L1, programmed cell death ligand 1; R, randomization; ROW, rest of the world; SCAC, squamous carcinoma of the anal canal; UK, United Kingdom.

POD1UM-303/InterAACT 2 (NCT04472429) is a global, multiregional, double-blind, randomized study investigating carboplatin–paclitaxel in combination with retifanlimab or placebo in patients with inoperable locally recurrent or metastatic SCAC not previously treated with systemic chemotherapy. Patients will receive up to six induction 28-day cycles (24 weeks) of carboplatin (area**-**under**-**the**-**curve 5 on day 1) and paclitaxel (80 mg/m^2^ on days 1, 8, and 15) as per SOC. Concurrently, retifanlimab 500 mg or placebo will be administered intravenously in a blinded fashion on day 1 of each 28-day cycle (every 4 weeks) for up to 13 cycles (six induction cycles plus seven retifanlimab or placebo cycles) in the absence of unacceptable toxicity, disease progression, withdrawal of consent, loss to follow-up, or premature discontinuation (**Figure 1**).

Crossover to open-label retifanlimab will be allowed for patients assigned to placebo upon verification of disease progression by blinded independent central radiographic review (BICR). Patients will be followed until all study participants have received 13 cycles (1 year) of retifanlimab or placebo, all assigned study drugs have been discontinued, or they experience second disease progression.

### 2.2 Patient selection

The key inclusion and exclusion criteria for the study are presented in **Table 1**. In brief, patients ≥18 years with inoperable locally recurrent or metastatic SCAC, measurable disease per RECIST v1.1 (25), and no prior systemic therapy received will be enrolled. Prior neoadjuvant or adjuvant therapy if completed ≥6 months before study entry is permitted. Patients who have received prior PD-(L)1-directed therapy, radiotherapy (with or without radiosensitizing chemotherapy) within 28 days of cycle 1 day 1, or palliative radiotherapy (≤30 Gy and not directed to the pelvic region) within 14 days of cycle 1 day 1 will be excluded. Tumor tissue biopsies collected during screening or archival biopsy samples will be evaluated for PD-L1 expression (central laboratory result is required for stratification and randomization), HPV, and microsatellite instability. Patients with well-controlled HIV infection (defined as CD4^+^ count ≥200/µl, undetectable viral load, and receiving antiretroviral therapy for ≥4 weeks before study enrollment) are eligible to be enrolled in the trial. Patients with impaired cardiac function, clinically significant cardiac disease, or history of organ or allogeneic stem cell transplant will not be eligible for the study.

Table 1Key inclusion and exclusion criteria.

**Table:** 

Inclusion criteria
18 years of age or older Histologically or cytologically verified, inoperable locally recurrent or metastatic SCAC No prior systemic therapy other than the following: •Chemotherapy administered concomitantly with radiotherapy as a radiosensitizing agent •Prior neoadjuvant or adjuvant therapy if completed ≥6 months before study entry Measurable disease per RECIST v1.1 as determined by local site investigator/radiology assessment and after any tissue collected during biopsy Adequate tissue and whole blood sample with the central testing result before randomization Biopsy for archival samples within 9 months before randomization ECOG performance status 0 to 1 If HIV-positive, then must be stable as defined by: •CD4^+^ count ≥200/μl •Undetectable viral load per standard of care assay •Receiving antiretroviral therapy (ART/HAART) for at least 4 weeks before study enrollment and have not experienced any HIV-related opportunistic infection for at least 4 weeks before study enrollment

**Table:** 

Exclusion criteria
Prior PD-(L)1-directed therapy Prior radiotherapy with or without radiosensitizing chemotherapy within 28 days of cycle 1 day 1 [or 14 days for palliative radiotherapy (30 Gy or less) that is not directed to the pelvic region] Receiving a live vaccine within 28 days of cycle 1 day 1 Known additional malignancy that is progressing or requires active treatment, or history of other malignancy within 3 years of study entry Active autoimmune disease requiring systemic immunosuppression in excess of physiologic maintenance doses of corticosteroids (>10 mg of prednisone or equivalent) Known active central nervous system metastases and/or carcinomatous meningitis Known active hepatitis A, B, or C virus infection Active infections requiring systemic therapy Impaired cardiac function or clinically significant cardiac disease

ART/HAART, antiretroviral therapy/highly active antiretroviral therapy; ECOG, Eastern Cooperative Oncology Group; HIV, human immunodeficiency virus; PD-(L)1, programmed cell death protein (ligand) 1; RECIST, Response Evaluation Criteria in Solid Tumors; SCAC, squamous cell carcinoma of the anal canal.

Enrolled patients will be stratified by PD-L1 expression (<1% versus ≥1%), extent of disease (locally recurrent versus metastatic), and region. Planned enrollment is approximately 300 patients worldwide, with 1:1 randomization to retifanlimab or placebo. The study is being conducted in Australia, Belgium, Denmark, France, Germany, Italy, Japan, Norway, Spain, the United Kingdom, and the United States.

### 2.3 Study endpoints and assessments

The primary study endpoint is PFS per RECIST v1.1 by BICR. The key secondary endpoint is OS; additional secondary endpoints include overall response rate (ORR), duration of response (DOR), disease control rate (DCR) by BICR, safety, and retifanlimab pharmacokinetics. A summary of the study endpoints including exploratory endpoints is presented in **Table 2**.

**Table 2:** Study endpoints.

Primary endpoint	PFS, defined as the time from the date of randomization until disease progression according to RECIST v1.1 by BICR or death from any cause
Key secondary endpoint	OS, defined as the time from the date of randomization until death from any cause
Secondary endpoints	ORR, defined as the percentage of patients having a CR or PR, according to RECIST v1.1 as determined by BICR Duration of response, defined as the time from the first documented response (CR or PR) according to RECIST v1.1 until disease progression as determined by BICR or death from any cause Disease control rate, defined as the number of patients maintaining either an objective response (CR, PR) or stable disease according to RECIST v1.1 as determined by BICR Number of patients experiencing AEs Number of patients discontinuing study drug because of AEs Population pharmacokinetics, including *C*_max_, *t*_max_, *C*_min_, and AUC_0–_ *t*
Exploratory endpoints	PFS 2, defined as the time from randomization to subsequent disease progression after initiation of new anticancer therapy (second disease progression), or death owing to any cause, whichever occurs first, as assessed by investigator review using RECIST v1.1 ORR crossover, defined as the percentage of patients in the crossover period having a CR or PR, according to RECIST v1.1 as assessed by investigator review using RECIST v1.1 Blood and/or tumor analytes, immune cell profiles, viral profiles, and other relevant markers Immunogenicity, defined as the occurrence of specific antidrug antibodies to retifanlimab HR-PRO assessments scheduled to align with tumor response HIV viral load and CD4^+^ counts in patients who are known to be HIV-positive

AE, adverse event; AUC_0–*t*_, area under the plasma or serum concentration–time curve from time = 0 to the last measurable concentration at time = *t*; BICR, blinded independent central radiographic review; *C*_max_, maximum observed plasma or serum concentration; *C*_min_, minimum observed plasma or serum concentration; CR, complete response; HIV, human immunodeficiency virus; HR-PRO, health-related patient-reported outcomes; ORR, overall response rate; OS, overall survival; PFS, progression-free survival; PR, partial response; RECIST, Response Evaluation Criteria in Solid Tumors; *t*_max_, time to maximum concentration.

Adverse events, including immune-related adverse events, will be coded by the Medical Dictionary for Regulatory Activities (MedDRA), with treatment-emergent adverse events (TEAEs) tabulated by preferred term and system organ class for all events, treatment-related events, and grade ≥3 events. Clinical safety laboratory analyses will include blood chemistries, hematology assessments, coagulation tests, endocrine function, and urinalysis. Additional laboratory monitoring in patients known to be HIV-positive will include CD4^+^ cell count and HIV viral load.

Several pharmacokinetic parameters will be assessed including maximum observed plasma concentration, time to maximum plasma concentration, minimum observed plasma concentration over the dose interval, and area under the plasma or serum concentration–time curve from time 0 to the last measurable concentration at time *t*.

### 2.4 Statistical analysis

#### 2.4.1 Sample size determination

At a one-sided overall 2.5% level of significance and true hazard ratio (HR) of 0.67 (under the alternative hypothesis), a total of 207 PFS events are required to have 83% power to reject the null hypothesis (HR = 1) using a log-rank test. Approximately 300 patients will therefore be randomized to the two treatment arms in a 1:1 ratio.

#### 2.4.2 Analysis of populations

The full analysis set includes all randomized patients and will be the primary population for all efficacy analysis. The safety population will include all randomized patients who received at least one dose of the study drug. The pharmacokinetic (PK) evaluable population will include all patients who received at least one dose of the study drug and provided at least one postdose sample (one PK measurement).

#### 2.4.3 Primary efficacy analyses

PFS will be assessed *via* BICR according to RECIST v1.1, with survival data analyzed by the Kaplan–Meier method and the estimated median with 95% confidence interval (CI) reported. There are no interim analyses for PFS.

#### 2.4.4 Key secondary efficacy analyses

OS data will be analyzed by the Kaplan–Meier method, and the estimated medians with 95% CI reported.

#### 2.4.5 Secondary efficacy analyses

ORR and DCR will be assessed *via* BICR according to RECIST v1.1. ORR and DCR including the respective 95% CIs will be determined. The odds ratio from the Cochran–Mantel–Haenszel test will be calculated for ORR. DOR data will be analyzed by the Kaplan–Meier method, and estimated medians with 95% CIs reported.

#### 2.4.6 Safety analyses

TEAEs will be tabulated by the MedDRA preferred term and system organ class for all events, related events, and grade ≥3 events. A data monitoring committee will meet at regular intervals to assess ongoing safety.

#### 2.4.7 Pharmacokinetic analyses

The collected PK data will be analyzed by standard population PK methods using appropriate software (e.g., NONMEM) or alternatively pooled with the data from other studies for a population PK analysis.

#### 2.4.8 Exploratory analyses

PFS 2 (PFS on the next line therapy) will be assessed using the same methods as PFS. ORR crossover (percentage of patients in the retifanlimab monotherapy crossover period having investigator-assessed CR or PR) will be determined if data permit. Translational analyses such as blood and/or tumor analytes, immune cell profiles and viral profiles, and assessment of patient-reported outcomes including the Quality of Life Questionnaire for Anal Cancer (QLQ-ANL27) are also planned.

## 3 Discussion

The combination of PD-(L)1 inhibitors with SOC chemotherapy has improved the outcomes for many patients, including other tumor types that are primarily driven by HPV. Specifically, improved survival has been demonstrated with the addition of pembrolizumab to SOC chemotherapy in recurrent or metastatic cervical cancer (15) and in combination with platinum–5-fluorouracil-based chemotherapy for the treatment of recurrent or metastatic HNSCC (26). Improvement in overall survival has also been shown with nivolumab in advanced HNSCC following progression on platinum-based chemotherapy (14) and with cemiplimab in the treatment of platinum-refractory cervical cancer (27).

The current preferred first-line regimen for unresectable locally advanced or metastatic SCAC is carboplatin–paclitaxel, with the reported PFS and OS of 8.1 and 20.0 months, respectively (20). Although new treatment options have been investigated (23, 24), there have been no approved therapies for SCAC in recent years. POD1UM-303 is one of the largest well-controlled, randomized trials of a novel therapeutic in locally inoperable or metastatic SCAC. It is anticipated that adding retifanlimab will increase PFS and OS over what can be achieved with a carboplatin–paclitaxel regimen. As observed with other immunotherapies, the results of this trial may have applicability to other HPV-driven tumor types adding further rationale and importance to this global phase III registration study.

## Trial status

Patient recruitment for POD1UM-303/InterAACT 2 began on 19 October 2020. The study protocol at the time of writing this report is INCMGA0012-303 Amendment 1 (Version 2), December 2021.

## Patient and public involvement

Patient and public input were solicited during the design of the study, including a patient advocacy board held to gain insight regarding participation in the trial.

## Ethics statement

POD1UM-303/InterAACT 2 is conducted in accordance with the International Council for Harmonisation of Technical Requirements for Pharmaceuticals for Human Use Guideline for Good Practice, the principles of the Declaration of Helsinki, and other applicable local ethical and legal requirements. The protocol and all amendments are reviewed and approved by the Institutional Review Boards or Independent Ethics Committees before the start of the study. Informed consent is obtained before patient participation in any study-related procedures. Study data and findings are planned to be presented at congresses and submitted for publication in peer-reviewed medical journals.

## Author contributions

All authors contributed to the study design, drafting and critical review of the manuscript, and provided approval of the final version to be published.

## Funding

This study is sponsored by Incyte Corporation (Wilmington, DE).

## Acknowledgments

The authors wish to thank the patients and their families, the investigators, and the site personnel participating in this study. The authors would like to thank Mark Cornfeld (Incyte Corporation, Wilmington, DE) for the critical review of this report. Medical writing assistance was provided by Matthew Bidgood, Ph.D., of Envision Pharma Group (Philadelphia, PA).

## Conflict of interest

SR reports advisory role or honoraria from Amgen, Celgene, and Shire and travel grants from Bayer, Celgene, and Incyte Corporation. MJ, JB, and CT report employment and stock ownership for Incyte Corporation. J-PS reports honoraria from AstraZeneca, Biogaran, Bristol Myers Squibb, Daiichi Sankyo, Eli Lilly, Gilead Sciences, Leo Pharma, Mylan, Myriad Genetics, Novartis, Pfizer, and Pierre Fabre; a consulting or advisory role for Merck Sharp & Dohme and Roche; and a grant from MSD Avenir.

## Publisher’s note

All claims expressed in this article are solely those of the authors and do not necessarily represent those of their affiliated organizations, or those of the publisher, the editors and the reviewers. Any product that may be evaluated in this article, or claim that may be made by its manufacturer, is not guaranteed or endorsed by the publisher.

## Glossary

**Table:**

AE	adverse event
ART/HAART	antiretroviral therapy/highly active antiretroviral therapy
AUC_0-_ *_t_*	area under the plasma or serum concentration-time curve from time = 0 to the last measurable concentration at time = *t*
BICR	blinded independent central radiographic review
CI	confidence interval
*C*_max_	maximum observed plasma or serum concentration
*C*_min_	minimum observed plasma or serum concentration
CR	complete response
DCR	disease control rate
DOR	duration of response
ECOG	Eastern Cooperative Oncology Group
HIV	human immunodeficiency virus
HNSCC	head and neck squamous cell carcinoma
HPV	human papilloma virus
HR	hazard ratio
HR-PRO	health-related patient-reported outcomes
ICB	immune checkpoint blockade
MedDRA	Medical Dictionary for Regulatory Activities
ORR	overall response rate
OS	overall survival
PD	progressive disease
PD-1	programmed death 1
PD-(L)1	programmed cell death (ligand) 1
PFS	progression-free survival
PK	pharmacokinetics
PR	partial response
RECIST	Response Evaluation Criteria in Solid Tumors
RT	radiotherapy
SCAC	squamous carcinoma of the anal canal
SOC	standard of care
TEAE	treatment-emergent adverse event
*t*_max_	time to maximum concentration
